# The Effects of the ‘Catabolic Crisis’ on Patients’ Prolonged Immobility after COVID-19 Infection

**DOI:** 10.3390/medicina58060828

**Published:** 2022-06-19

**Authors:** Titus David Moga, Carmen Delia Nistor-Cseppento, Simona Gabriela Bungau, Delia Mirela Tit, Anca Maria Sabau, Tapan Behl, Aurelia Cristina Nechifor, Alexa Florina Bungau, Nicoleta Negrut

**Affiliations:** 1 Faculty of Medicine and Pharmacy, University of Oradea, 410073 Oradea, Romania; titusmoga@gmail.com; 2 Department of Psycho Neuroscience and Recovery, Faculty of Medicine and Pharmacy, University of Oradea, 410073 Oradea, Romania; lnm_n10@yahoo.com; 3 Doctoral School of Biological and Biomedical Sciences, University of Oradea, 410087 Oradea, Romania; mirela_tit@yahoo.com; 4 Department of Pharmacy, Faculty of Medicine and Pharmacy, University of Oradea, 410028 Oradea, Romania; 5 Department of Physical Education, Sport and Physical Therapy, Faculty of Geography, Tourism and Sport, University of Oradea, 410087 Oradea, Romania; sabauancamaria@yahoo.com; 6 Chitkara College of Pharmacy, Chitkara University, Rajpura 140401, India; tapanbehl31@gmail.com; 7 Analytical Chemistry and Environmental Engineering Department, Polytechnic University of Bucharest, 011061 Bucharest, Romania; aureliacristinanechifor@gmail.com; 8 Medicine Program of Study, Faculty of Medicine and Pharmacy, University of Oradea, 410073 Oradea, Romania; pradaalexaflorina@gmail.com

**Keywords:** bone, muscle, osteoporosis, sarcopenia, physical performance, prolonged immobilization, DEXA determination

## Abstract

*Background and Objectives*: Quarantine, isolation and bed rest associated with COVID-19 infection favored the loss of muscle and bone mass, especially in elderly patients. The current study aims to compare the presence of sarcopenia and osteoporosis in patients with a recent (one month) history of SARS-CoV-2 infection versus the general population. *Materials and Methods*: A cross-sectional study was conducted in which 157 patients were enrolled, divided into two groups, comparable in structure. The COVID-19 group (group C) consisted of 86 patients who were diagnosed with SARS-CoV-2 respiratory infection within the last 30 days. The non-COVID-19 group (group NC) consists of 71 patients who had no clinical signs of respiratory infection and were not quarantined/hospitalized in the last 3 months. Muscle strength, incidence of sarcopenia (using SARC-F score) and osteoporosis (DEXA determination) and physical performance (SPPB score) in the two groups were assessed and compared. *Results*: No statistically significant differences were found between the SPPB scores of the C group versus the NC group. Statistically significant differences were found in the evaluation of three parameters included in the SARC-F score. Patients in the C group had difficulties in standing up from a chair (*p* = 0.009) and climbing stairs (*p* = 0.030) due to lower muscle strength (*p* = 0.002) compared with patients in the NC group. No correlation of the SARC F and SPPB scores with the T score values obtained by osteo-densitometry was found. *Conclusions*: The sudden and significant reduction in physical activity, through various measures taken in the general population during the pandemic, led to an increased incidence of sarcopenia, both in patients who did not have COVID-19 infection and among those quarantined/hospitalized for this condition.

## 1. Introduction

Sarcopenia is defined as a decrease in muscle mass, strength and function [1] and is an age-related health problem, especially as the average life expectancy of the population has increased [2]. The definition of sarcopenia continues to evolve from an observational phenomenon to a different diagnostic approach. Studies on the prevalence of sarcopenia in the world have shown that the results are insufficiently comprehensive [3]. It is generally accepted that the onset of sarcopenia is multifactorial and may be related to hormonal decline, muscle fiber decline, chronic inflammatory status, inadequate dietary intake, and chronic diseases causing reduced physical activity or causing malabsorption [4,5]. Sarcopenia is associated with aging and may be seen in geriatric patients. However, it can also be seen in younger patients. After the age of 40, a slight decrease in muscle mass is observed, which becomes more pronounced with advancing age [6]. The literature describes a loss of muscle mass of about 8% in 10 years after the age of 40 and 15% after the age of 70. Sarcopenia affects about 30% of people over 60 and over 50% of people over 80. The number of people worldwide aged over 60 was estimated at 600 million in 2000, a figure that is expected to rise to 1.2 billion by 2025 and 2 billion by 2050 [7]. It is estimated that 20% of the general population is affected, a percentage that is still increasing, and by 2045 sarcopenia could affect 63% of the population; 8–12% of men and 8–13% of women are affected [8]. In results obtained by bioelectrical impedance analysis for muscle mass, prevalence was higher among European than Asian individuals (19% vs. 10% in men; 20% vs. 11% in women) [3].

Sarcopenic changes are characterized by qualitative and quantitative changes in muscle fibers, alpha neurons, protein synthesis and production of anabolic and sex hormones [1]. All these changes lead to decreased muscle function, increased frailty, and loss of independence. In the elderly, selective atrophy of type II fibers occurs, and, due to neurodegeneration of skeletal muscle fibers, recruitment of type II fibers during resistance exercise (RE) decreases [9]. Associated with this mechanism are additional mechanisms, induced anabolic resistance, insulin resistance (IR), mitochondrial dysfunction and associated oxidative stress [10]. Another mechanism that may contribute to anabolic resistance in older populations is reduced skeletal muscle capillarization, which may reduce the hypertrophic effect of RE. Drug treatment has not sufficiently defined its efficacy. Prophylactic or curative interventions mainly target nutrition and exercise [11]. These have an impact on physical performance, with improvements in walking speed, standing up from a chair, etc. [2]. 

Considerable research has been conducted on the correlation between sarcopenia and autoimmune conditions such as rheumatoid arthritis. Chronic inflammation is thought to contribute to sarcopenia, even though the pathophysiology of sarcopenia in autoimmune disorders has not been fully understood. Furthermore, the pathogenesis appears to vary according to the specific underlying condition [12,13].

Osteoporosis (OP) is characterized by a reduction in bone mass associated with changes in the microarchitecture of bone tissue. This leads to increased bone fragility and an increased risk of fracture. In the case of OP, the importance of nutritional factors of calcium and vitamin D is assessed. The risk of developing a fracture due to OP 10 years after onset is 40% [14].

The declaration of the COVID-19 pandemic has led to the institution of physical activity restrictions and lifestyle changes for the entire population. The new habits have led to increased sedentarism, increased food intake, decreased muscle mass, and increased body fat, with implications on pre-existing pathologies or favoring the onset of diseases (diabetes, cardiovascular disease, depression) [10]. Moreover, the quarantine, isolation and bed rest associated with COVID-19 infection favored the loss of muscle and bone mass, especially in elderly patients. Moderate and severe forms required isolation and hospitalization of the patient for often prolonged periods due to complications or the need for specific treatment in the intensive care unit [15]. Published studies show an average length of hospitalization of 11 days for patients with severe acute respiratory syndrome coronavirus 2 (SARS-CoV-2) [10]. Associated with this, post-infective fatigue syndrome has been reported in 46% of patients with COVID-19, with a variable duration of 16–20 weeks from the onset of symptoms [16]. These are associated with anxiety and depression, which favor lack of physical activity with loss of muscle mass and changes in eating behavior, favoring weight gain and metabolic syndrome. Due to the dramatic change in lifestyle, physical activity in general, but especially in the case of isolation, quarantine or hospitalization during the COVID 19 pandemic, the body also undergoes a series of hormonal changes, expressed by a reduction in testosterone, estrogen, and growth hormone, which play a role in bone remodeling, favoring the appearance of OP [10].

Osteosarcopenia is a new syndrome defined by osteoporosis and sarcopenia [17], responsible for the onset of disability in the elderly, has been shown to be correlated with social isolation and decreased physical activity. Sarcopenia and osteoporosis are two pathologies that are associated with the elderly. A study of a sample of 679 European men showed a three-fold higher incidence of OP in patients with sarcopenia. Over the last ten years, the mechanical interrelationship between bone and muscle tissue has been studied [18]. There is a close relationship between the two tissues, both being controlled by the endocrine system [19]. Reduced secretion of anabolic hormones, increased activity of inflammatory cytokines, anabolic molecules releasing myokines and osteokines, and reduced physical activity are some common mechanisms for sarcopenia and osteoporosis [20]. Diagnosis of osteosarcopenia is based on the number of annual falls, fracture history, clinical features of osteoporosis (spinal subsidence kyphosis) and sarcopenia (i.e., decreased muscle strength, physical dysfunction, falls) [21]. More and more publications support evidence for the coexistence of loss of bone and muscle substance under normal aging or pathological conditions [17,18].

The current study aims to compare the presence of sarcopenia and osteoporosis in patients with a recent (one month) history of SARS-CoV-2 infection versus the general population. As far as we know, very few papers have been published worldwide on this specific topic, none of them about Romanian patients.

## 2. Materials and Methods

### 2.1. Study Design

A prospective cross-sectional study was carried out between February 2020 and February 2021, at the Medical Treatment and Rehabilitation Centre, Baile 1 Mai, Ceres Hotel, Bihor County, Romania, in order to compare the presence of sarcopenia and osteoporosis in patients with a recent (one month) history of SARS-CoV-2 infection vs. the general population. A total of 1256 consecutive hospitalized patients with various musculoskeletal disorders (neurological or degenerative) were evaluated for being included in the study. The selection of subjects followed the formation of two groups:

- Non-COVID-19 group (group NC) of patients who had no clinical signs of respiratory infection, had not been quarantined/hospitalized in the last 3 months, and at the time of evaluation, had no positive SARS-CoV-2 antibodies (immunoglobulin M or G) (rapid chromatographic immunoassay, Hoffmann-La Roche Ltd., Basel, Switzerland).
- COVID-19 (group C) group of patients who were diagnosed with SARS-CoV-2 respiratory infection within the last 30 days. The diagnosis was based on confirmation of COVID-19 using a single positive test, which highlights the RNA/antigen of the virus in the upper respiratory tract specimens (nasopharyngeal and oropharyngeal) using the real-time polymerase chain reaction/rapid chromatographic immunoassay.

### 2.2. Inclusion/Exclusion Criteria

Patients with osteopenia and osteoporosis (T score < −1.1) and those with low muscle strength (SARC-F score ≥ 4) were included. Exclusion criteria considered were as follows: T score > −1.1, patients with a history of malignant tumors, organ failure, presence of joint pathology limiting assessments and refusal to participate in the study. 

**Figure 1:**
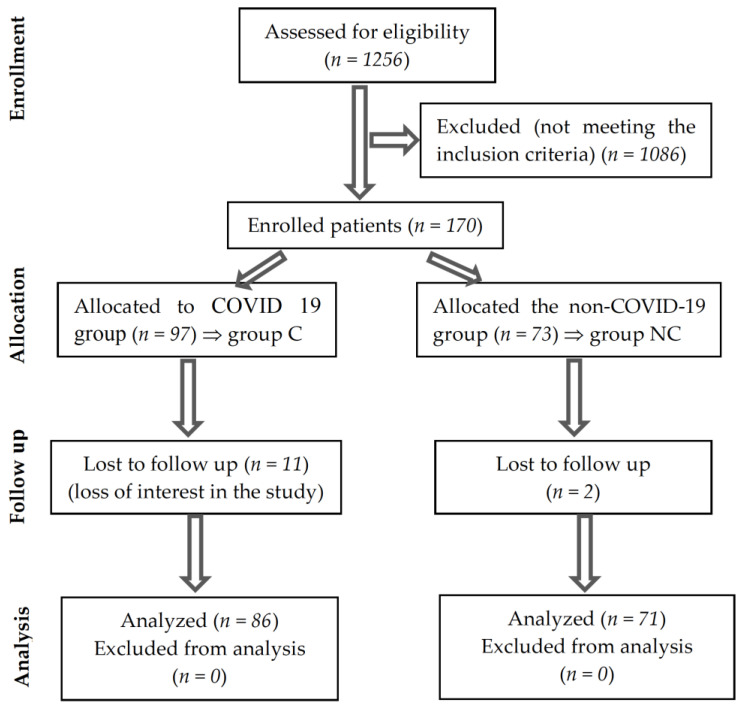
CONSORT flow diagram of the study.

A total of 170 patients were declared eligible during the 12 months studied. During the period of study, 13 cases could not be followed up because they did not show up for all the assessments; Figure 1 summarizes the information provided above. The sample was relevant with a 95% probability.

### 2.3. Sample Size

The sample size of subjects included in the study was calculated considering the total number of patients who visited the outpatient clinic during the study period. To calculate the sample size, we considered the following variables: p—the probability of occurrence of the phenomenon, 0p1, q—counter-probability, q = 1-p, t—probability factor, x—the error limit, N—the volume of the community.

To determine the sample of cases, we used the formula: *n* = t^2^ pq/(x^2^ + t^2^ pq/N). The formula is valid for studies in which the characteristic followed is an alternative (in our case healthy–sick). The value of n is maximum if the product of pq is maximum, i.e., when p = q = 0.5. The probability of 95% corresponds to a value of t = 1.96. A limiting error of 0.1 was set. If *N* is large, above 10,000 (in our case *N* = 1256), the ratio t^2^ pq/N is neglected. The value obtained by the above formula is *n* = 85.

### 2.4. Study Tools

**Figure 2:**
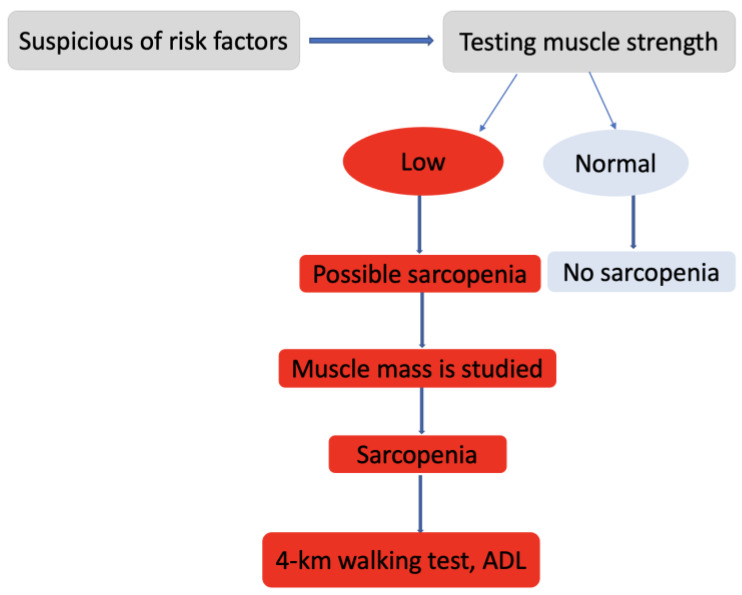
Diagnostic algorithm for sarcopenia in the elderly (EWGSOP). ADL, daily activity living.

All evaluated patients had their bone density determined by an osteo densitometer/DXA device (MEDIX 90, EIMSA ELECTRONICA Y MEDICINA, S.A., Montpellier, France), physical performance by Strength Assistance with walking Rising from a chair Climbing stairs-Falls (SARC-F) questionnaires and Short Physical Performance Battery (SPPB) tests. Measurements were made with the JAMAR Pneumatic dynamometer (Sammons Preston, Biobank, Bolingbrook, IL, USA) to determine the maximum voluntary strength of the right and left upper limb muscles. In this study, we applied the diagnostic algorithm for sarcopenia suggested by The European Working Group on Sarcopenia in Older People (EWGSOP) in 2019 [18] (Figure 2). It is a simple and quick diagnostic algorithm that can be used in clinical practice. 

The SARC-F questionnaire contains five questions assessing strength, gait, transfer from chair or bed, stair climbing, and falls [22]. It is considered one of the best tools available for use in primary care for raising awareness of sarcopenia diagnosis, having high specificity but low sensitivity for classification of sarcopenia [23]. Each component is scored from 0 to 2 points, giving an overall SARC-F score between 0 and 10 points (Table 1). 

A score ≥ 4 points is reported to be predictive of sarcopenia, recommending a detailed assessment to determine sarcopenia. The SPPB questionnaire measures physical performance by testing the ability to get up from a chair, balance, and walking speed. These tests focus on lower extremity function, as the latter has been shown to correlate with mobility and the patient’s disability and is accentuated after hospitalization or institutionalization [24]. Values between 0 and 6 show low performance, and those between 7 and 9 show intermediate performance. SPPB scores between 10 and 12 correspond to high physical performance. Poor physical function was defined as follows: gait speed < 1.0 m/s, time in the 5-contracted chair stand test (5CST) ≥ 12 s and a score value ≤ 9. Walking speed is measured as comfortable walking speed on flat ground of 4 m. The 5CST time is the time it takes to stand up from a seated position and sit down as quickly as possible five times (test performed without upper limb support). SPPB is a physical functioning test comprising standing balance, gait speed and 5CST time; each item is scored from 1 to 4, with the final score ranging from 0 to 12 points (Table 2), according to published data [25], with higher scores indicating better physical functioning [22].

Using the JAMAR dynamometer, the maximum voluntary force (which is the maximum force that can be maintained during an isometric contraction for a duration of 3–4 s) was measured. The measurement is performed with the patient in a seated position; it is repeated 3 times, and the maximum value determined is chosen. A variety of grip strength thresholds have been proposed to characterize low muscle strength, ranging from 16 to 20 kg for women and 26 to 30 kg for men for the upper limbs [24]. In the study, we considered the reference of 18 kg for women and 28 kg for men (the arithmetic means of the reference ranges). Isometric and/or isokinetic strength measurements can be performed. Both upper limbs were measured by isometric measurements.

Bone mineral density (BMD) was measured at the femoral neck and at the level of the lumbar L2–L4 spine. Osteoporosis is defined by T-score values, measured by osteo densitometry, <−2.5; osteopenia is characterized by T-score values between −1.1 and −2.5.

### 2.5. Ethical Approval

The study was approved by the institutional review board of the Medical Treatment and Rehabilitation Centre, Baile 1 Mai, Ceres Hotel, Bihor County, Romania (3918/16.11.2020). The research was conducted in compliance with the Declaration of the World Medical Association of Helsinki [26]. Participation in the study was voluntary. Written informed consent was obtained from all participants as a tool for accurate information processing, improved decision-making capacity, collection, and processing of databases.

### 2.6. Statistical Analysis

Statistical analysis was generated using the Statistical Package for the Social Sciences, version 28. Means, standard deviations and tests of statistical significance were determined. The calculation of the *p*-value was realized using Student’s t-test and chi-square test. The statistical significance was considered for *p*-values < 0.05.

The values obtained for different parameters were considered primary data for determining the correlation coefficient. To obtain an indicator independent of the units of measurement, the Bravais–Pearson correlation coefficient (r) was used. The interpretation of the coefficient r is valid only for cases with *p* < 0.05.

## 3. Results

The two groups did not present statistically significantly different characteristics in terms of the environment of origin, risk factors or sex (Table 3). The evaluated risk factors were identified in 52 (60.46%) of the patients included in group C and in 38 (53.52%) in group NC, *p* = 0.140. The most common risk factor was coffee user (26.74% for group C and 18.31% for NC group), followed by smoking (24.42% C vs. 15.49% NC).

**Figure 3:**
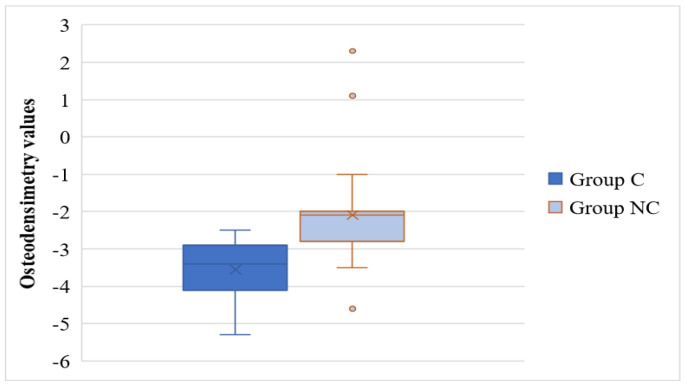
Distribution of T score values in the study groups. C, COVID-19 patients; NC, non-COVID-19 patients; o, mild outliers.

Based on the diagnosis realized after the DEXA examination (BMD osteopenia/osteoporosis), the prevalence of osteoporosis was 98.83% for the C group and 42.25% for the NC group, *p* < 0.001. Group C patients had statistically significantly lower values of bone mineral density compared to group NC (T score = −3.55 ± 0.72 vs. T score = −2.09 ± 1.06, *p* < 0.001), as it is depicted in Figure 3.

No statistically significant differences were found between the SPPB scores of the C group versus the NC group on all three domains assessed. Most patients had low-performance values of SPPB scores, but no statistically significant differences between the two groups were found (*p* = 0.919) (Table 4).

The values of SARC-F for the C group were statistically significantly lower than in the NC group (0.414). The statistically significant differences are in the evaluation of three parameters included in the SARC-F score. Patients in the C group presented difficulties in standing up from the chair (*p* = 0.009), climbing stairs (*p* = 0.030) due to lower muscle strength (*p* = 0.002) compared with patients in the NC group. The number of patients with a value of SARC-F score of at least four did not differ statistically significantly between the two groups. The number of subjects with low, intermediate, or high performance was not statistically significant between group C and group NC (Table 4).

The correlation index between the SPPB score and the values of bone mineral density showed was r = −0.028, *p* = 0.400, which indicates the lack of correlation between the two variables in the C group. No correlation was found in group C either in the case of the SARC-C score and bone mineral density values (r = −0.179, *p* = 0.051).

**Figure 4:**
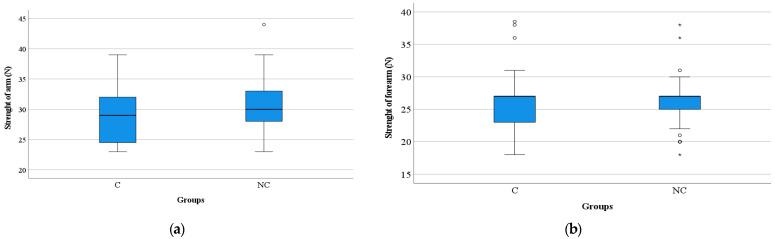
Distribution of muscle strength values in the study groups: (**a**) in arm, (**b**) in forearm. C, COVID-19 patients; NC, non-COVID-19 patients; N, Newton; *, extreme outliers; o, mild outliers.

Significantly higher values of muscle strength were found in the arm level in group NC compared to C (arm: 30.48 ± 4.48 vs. 28.63 ± 4.24, *p* = 0.009) but not in the forearm level (26.07 ± 3.15 vs. 25.79 ± 3.65, *p* = 0.606) (Figure 4). 

The number of cases that had low arm and forearm strength did not differ statistically significantly between the two groups, according to the patient’s gender (Table 5).

## 4. Discussion

The multidimensional concept of physical performance has been updated, and a new definition has been developed: “an objectively measured function related to mobility”. Measurements mostly refer to the patient’s ability to move and transfer. Decreases in physical performance may be evident before the onset of the inability to perform daily activities.

In older adults, bed rest facilitates a reduction in protein synthesis and an accelerated loss of muscle mass, strength, power, and functional capacity. The negative metabolic and morphological consequences of bed rest are exacerbated by pre-existing sarcopenia. It has been shown that muscle mass is lost after 30 years, 3–8% per decade [27]. Sarcopenia affects approximately 30% of people over 60 years of age, with a maximum in people over 80 years of age [28]. In the study, the mean age of the patients is 66.56 ± 7.49, respectively 66.79 ± 7.61, and 83% of the subjects included in the study are over 60 years old, with no significant differences between groups.

Mean values of body mass index (28.42 ± 4.78, 28.07 ± 4.69) are above normal values (18.50 and 24.99). Only 30.5% of the patients had normal weight values, and the rest of the patients had above normal values, being overweight, obesity grade 1 and 2. In the evaluated groups, there were no patients with BMI < 18, which is considered to have an increased prevalence of limitation of function due to reduced muscle mass. Functional consequences are more accurately described by the term sarcopenic obesity than decreased muscle mass [1]. Obesity is a risk factor for limited mobility and reduced physical activity. The study conducted by Molfino et al. on a group of 25 patients with an average age of 67 years (2004) supports the idea that the risk of disability is increased when obesity and sarcopenia are associated [29,30]. The same study supports a decrease in muscle strength in sarcopenic obese patients. Our study shows that all patients included in the two groups have low performance (SPPB score ≤ 9), without significant differences between groups; sarcopenia (SARC-F score ≥ 4) is present in 32 patients (37.21%) in group C and in 23 patients (32.39%) in group NC (*p* = 0.224). 

Lifestyle changes, reduction in physical activity through prolonged hospitalization or quarantine associated with prolonged bed rest, and dietary changes were factors favoring the accentuated loss of bone and muscle mass. The mean value obtained indicates the presence of OP in group C (−3.55 ± 0.72), while the mean value of the T score in group NC indicates osteopenia. No significant differences in the presence of kyphosis, scoliosis, and fractures secondary to osteoporosis were found between the two groups. A systematic review of electronic databases, published in 2021, identified an increased incidence of hip, humerus and radiocarpal fractures and vertebral subsidence (consequences of OP) associated with an increased risk of death/hospitalization due to SARS-CoV-2 infection in women [31].

The study of 114 patients (2021) demonstrated an increased incidence of vertebral impingement (36%) in patients admitted for severe pneumonia with SARS-CoV-2, compared to the general population (18–26% in women and 8–20% in men) [32]. Mortality was higher in those with severe vertebral fractures (60%) compared to those with moderate or mild vertebral fractures (7% and 24%, respectively). The presence of SARS-CoV-2 infection triggers the pathophysiological mechanism of increased pro-inflammatory cytokines due to prolonged immobilization, which favors bone resorption, thus supporting the idea of osteoporosis predisposition in patients with this pathology. SARS-CoV2 infection has been detected in 13% of patients with hip fracture, and the risk of death in this case increases seven-fold [31].

A meta-analysis performed on 68 studies (2021) that included a patient number of 98,502 looked at the association of socio-demographic, behavioral (nutrition, smoking, physical activity), and associated disease (including osteoporosis) factors with sarcopenia among the general population. Only six studies have reported decreased bone density and support that OP is a risk factor associated with sarcopenia in the elderly population [33]. Sarcopenia is significantly related to osteopenia and osteoporosis, as demonstrated in a study of 3077 volunteers over 65 years of age, regardless of associated pathology (Lee et al., 2021). Sarcopenia was determined in 1230 (39.9%), of whom 41.8% were men (*p* = 0.133). Osteopenia was present in 1402 evaluated subjects (44.0%), of which 53.1% (*n* = 750) were male (*p* < 0.001); osteoporosis was diagnosed in 1156 patients (39.9%), of which 990 were female (59.9%, *p* < 0.001), with higher prevalence in men (54.9% vs. 67.9% in women) [34]. None of the studies demonstrated an association of smoking with sarcopenia [33].

Evidence shows a statistically significant difference in the sarcopenic index, which assesses muscle strength, gait assistance, chair lifting, stair climbing and falls between the two groups studied. The mean value determined for group C (5.04 ± 2.17) is significantly higher than the mean value determined in patients who did not have COVID-19 infection. Significant differences occur in the measurement of muscle strength, rising from the chair and climbing stairs. Walking assistance and incidence of falls is comparable for the two groups, explained by the homogeneity of the groups (age, sex, risk factors). Hospitalization and quarantining resulted in accelerated loss of bone and muscle mass. Kirwan et al. show that in 2 days of immobilization, approximately 1.7% of muscle volume is lost, and in 7 days, a loss of 5.5% is reached [11]. A review of the literature [35] on prospective cohort studies demonstrated the association between sarcopenia and falls in older adults.

The determination carried out using the SPPB questionnaire for the assessment of lower limbs (ability to stand up from the chair, balance, walking speed) does not show significant differences between the two groups (*p* > 0.05), explained by the fact that during the pandemic everyone’s lifestyle was affected by the closure of sports halls and rehabilitation centers (imposed by the social distancing). The values obtained in both groups are ≤6 points, indicating low performance in more than half of the subjects recruited in both groups (58.14% in group C and 69.01% in group NC); the remaining patients scored between 7 and 9, interpreted as intermediate low performance. Score ≤ 9 is associated with mobility-related disability [36].

The World Health Organization’s recommended physical activity of 150 min/week before the pandemic, consisting of aerobic exercise 2 days/week, or daily walks of 30 min/day, could not be met during the pandemic. The activity of the population, especially the elderly, was significantly restricted. Because of this, the number of patients with a SARC-F score ≥ 4 did not differ statistically significantly between the two groups (Table 4).

A prospective study of 117 healthy male patients aged 53–65 years, assessed according to the EWGSOP2 definition of sarcopenia (muscle strength, physical performance), before and after the acquisition of COVID-19 infection, showed that 27.35% were sarcopenic and 72.64% of participants were non-sarcopenic, at baseline. At reassessment, 26.49% developed sarcopenia after COVID-19 infection. Furthermore, SARC-F score values were higher in patients who initially had sarcopenia compared to non-sarcopenic patients. These data indicate a potential crosstalk between viral infection and sarcopenia phenotype [37]. In comparison, our study shows an incidence of sarcopenia of 32.39% in patients (group NC) who did not have a disease restricting their physical activity and 37.21% in group C, with no statistically significant differences (*p* = 0.224), explained by the marked reduction in physical activity among the whole population.

A study of 680 patients over 70 years of age over 18 months on the association between bed rest and functional decline demonstrated a relationship between time spent on bed rest and the extent of functional decline with decreased mobility, physical and social activity, in the performance of ADLs [38]. The incidence of moderate sarcopenia among bedridden American men is 71% and 42% among women (≥65 years) [27]. In the study conducted (it is understood as ours), the overall incidence of sarcopenia is 20% in adults over 70 and over 50% in bedridden individuals over 80. The severe form of sarcopenia, which is responsible for accentuated disability, is more frequently detected in men (17% versus 11% in women).

Marked reduction in physical activity imposed during the pandemic defines “catabolic crisis” [27] occurring as a particular pattern of sarcopenia that is favored by periods of prolonged bed rest or hospitalization. With an average hospital stay of 12 days in the case of COVID-19 infection, muscle mass decreases [36]. This could explain the lack of correlation between the occurrence of sarcopenia and osteoporosis in the groups studied, although it has been shown that most fractures occur due to falls, and a link between osteoporosis and sarcopenia is established [21].

Regarding the assessment of muscle strength at the arm level, using the dynamometer, significant differences were detected between the two groups, differences that are not found in the assessment of forearm muscle strength but without significant differences between the two groups (Table 5). The strength measured in the arms and forearms is low in men in both groups, which indicates an increased susceptibility to sarcopenia in men. This correlates with data from the literature [27]. Inactivity mainly affects lower limb muscles. In healthy young adults, the losses are low, and the average determined is 2.6 kg (force measured by dynamometer) after 119 days of bed rest and 0.4 kg in 28 days. We found no studies comparing mass loss and muscle strength in young versus older adults. A linear calculation performed shows a three to six times greater loss in the elderly [27].

Resistance exercise is considered to be the most important solution for the prevention of sarcopenia in the elderly, but the positive effect on osteopenia/osteoporosis in men has yet to be confirmed [39]. For increasing physical performance, walking on flat ground with a progressive increase in distance is recommended. It takes at least 3 months of training to see significant results. It will influence the increase in immunity (it favors exposure to ultraviolet radiation, leading to an increase in vitamin D synthesis), the maintenance of weight and, implicitly, the patient’s quality of life. It is proven that walking three times a week [17] reduces the risk of depression in women, especially important in the context of isolation during the pandemic. Resistance training programs have also been shown to be effective in increasing strength, but physical capacity, i.e., walking speed and standing, has improved modestly [1].

Several studies report acute sarcopenia associated with COVID-19 infection [40,41,42]. In these studies, particular emphasis is placed on the assessment of muscle strength, physical performance, and balance as well as treatment monitored by a multidisciplinary team. The results of the study indicate the presence of sarcopenia (SARC-F score ≥ 4) in 37.21% (group C) and 32.39% (group NC), respectively.

D. Levy et al. demonstrate the incidence of sarcopenia in 16% (22) of 139 patients evaluated 3 months after the onset of COVID-19 infection [43]. The only risk factor identified was prolonged hospitalization due to viral infection. Evaluation at 6 months showed that 16 of the 22 patients who were diagnosed with this disease were cured. In the study, the results showed a similar incidence of sarcopenia in the two groups (37.21% in group C and 32.39% in group NC), which can be explained by the presence of sarcopenia prior to the infection, especially as the majority of patients included in the study were over 60 years of age when the loss of muscle mass was more pronounced.

This study supports the interaction between osteosarcopenia and COVID-19 infection, as well as their potentiation, as do other publications [37]. Assessment of sarcopenia in individuals with risk factors, symptoms and/or conditions that put them at risk of disability will become particularly important in the near future [23]. The onset of sarcopenia is insidious, but its progression can be greatly accelerated by physical inactivity and poor nutrition. The most important aspects affected are assistance walking and climbing stairs, explained by decreased muscle strength caused by the “catabolic crisis”, the new model of sarcopenia. Public health policies can follow the establishment of home strategies based on resistance exercise [24], higher protein intake and vitamin and Ca supplements to modify lifestyle and avoid a post-COVID-19 rehabilitation crisis. Organized physical activity has been shown to prevent osteosarcopenia [17]. Such strategies may also serve as useful preventive measures to reduce the likelihood of sarcopenia in general and in future periods of isolation.

### Strengths and Limitations of the Study

This study is the first in Romania to comparatively evaluate the presence of sarcopenia and osteoporosis in patients with a recent (one month) history of SARS-CoV-2 infection versus the general population. The obtained results highlight the presence of the catabolic crisis in patients with prolonged immobilization after COVID 19 infection and an increased incidence of sarcopenia among the general population, determined by the limitations imposed by the pandemic, regardless of the associated diseases. Moreover, this research can be a starting point for thinking/developing/implementing optimal public health policies on sarcopenia prevention programs in isolation conditions imposed by various emergencies or disasters.

The main limitation of the present study is the cross-sectional character of the study; there were no assessments of physical performance and presence of sarcopenia, hormonal determinations (testosterone, estrogen) or determinations of bone densitometry, muscle strength, or COVID-19 infection staging prior to the study. The study design hinders the interpretation of results and only indicates a potential relation between COVID-19 and osteosarcopenia.

## 5. Conclusions

The sudden and significant reduction in physical activity, through various measures taken in the general population during the pandemic, led to an increase in the incidence of sarcopenia, both in patients who did not have COVID-19 infection and among those quarantined/hospitalized for it. Sarcopenia and osteoporosis have an increased incidence in the general population, but the values that define osteosarcopenia are more important in patients in the positive COVID-19 group due to isolation or prolonged bed rest due to disease or due to increased residual asthenia. The most important aspects affected are walking and climbing stairs, explained by the decrease in muscle strength caused by the “catabolic crisis”, the new model of sarcopenia. It is imperative to implement a physical training program to prevent these pathologies.

Future research directions require further studies to establish, first and foremost, the most appropriate design for studies on the same/similar topic, as well as to develop an optimized strategy for the prevention and treatment of osteosarcopenia.

## Figures and Tables

**Table 1:** SARC-F score.

Component	Question	Scoring
Strength	How much difficulty do you have in lifting and carrying 4.5 kg?	None = 0 Some = 1 A lot or unable = 2
Assistance in walking	How much difficulty do you have walking across a room?	None = 0 Some = 1 A lot, use aids, or unable = 2
Rise from a chair	How much difficulty do you have transferring from a chair or bed?	None = 0 Some = 1 A lot, or unable without help = 2
Climb stairs	How much difficulty do you have climbing a flight of 10 stairs?	None = 0 Some = 1 A lot or unable = 2
Falls	How many times have you fallen in the past year?	None = 0 1–3 falls = 1 ≥4 falls = 2

**Table 2:** Short physical performance battery protocol and score sheet (SPPB) [25].

Component	Question	Scoring Points
Balance	Held for 10 s	4
	Held for 3 to 9.99 s	3
	Held for <than 3 s	2
	Not attempted	1
For 4-Meter Walk	If time is >8.70 s	1
	If time is between 6.21 and 8.70 s	2
	If time is between 4.82 and 6.20 s	3
	If time is <4.82 s	4
Chair Stand Test	Participant unable to complete 5 chair stands or completes stands in >60 s	
	If chair stand time is ≥16.70 s	1
	If chair stand time is between 13.70 and 16.69 s	2
	If chair stand time is between 11.20 and 13.69 s	3
	If chair stand time is ≤11.19 s	4

**Table 3:** Baseline characteristics of the groups.

Parameter	Group C	Group NC	*p*
Age, M, SD	66.56 ± 7.49 (86)	66.79 ± 7.61(71)	0.853 *
Age groups			
<60, M, SD (*N*)	55.67 ± 4.43 (15)	55.50 ± 4.10 (12)	0.920 *
60–70, M, SD (*N*)	65.45 ± 2.66 (47)	65.46 ± 3.92 (39)	0.980 *
>70, M, SD (*N*)	75.96 ± 3.26 (23)	76.15 ± 3.34 (20)	0.849 *
Female, N (%)	47 (54.65%)	41 (57.74%)	0.522 **
Rural area, N (%)	40 (46.51%)	35 (49.30%)	0.563 **
BMI, M, SD	28.42 ± 4.78	28.07 ± 4.69	0.647 *
Smoker, N (%)	21 (24.42%)	11 (15.49%)	0.077 **
Alcohol user, N (%)	8 (9.30%)	14 (19.72%)	0.200 **
Coffee user, N (%)	23 (26.74%)	13 (18.31%)	0.095 **
PMH, N (%)			
Kyphosis	81 (94.19%)	68 (95.77%)	0.286 **
Scoliosis	54 (62.79%)	43 (60.56%)	0.264 **
Bone fractures	18 (20.93%)	13 (18.31%)	0.369 **

M, mean value; SD, standard deviation value; *N*, total number; BMI, body mass index; PMH, past medical history; *p* values, statistical significance (*, *t*-test; **, chi-square test).

**Table 4:** SPPB and SARC-F score values of the groups.

Parameter	Group C	Group NC	*p*
SPPB			
Score values, M, SD	6.00 ± 2.01	6.27 ± 2.05	0.414 *
Low performance, values 0–6, *N* (%)	50 (58.14)	49 (69.01)	0.919 **
Intermediate performance, values 7–9, *N* (%)	36 (41.86)	22 (30.99)	0.066 **
High performance, values 10–12, *N* (%)	0 (0)	0 (0)	-
Balance, M, SD	1.58 ± 0.61	1.75 ± 0.53	0.063 *
4-m walk, M, SD	2.02 ± 0.92	1.99 ± 0.92	0.798 *
Chair standing, M, SD	2.43 ± 1.44	2.54 ± 1.45	0.648 *
SARC-F			
Score values, M, SD	5.04 ± 2.17	4.24 ± 2.43	0.035 *
Sarcopenie, values ≥ 4, *N* (%)	32 (37.21)	23 (32.39)	0.224 **
Strength, M, SD	1.25 ± 0.67	0.92 ± 0.63	0.002 *
Assistance walking, M, SD	0.71 ± 0.63	0.68 ± 0.65	0.773 *
Rise from a chair, M, SD	1.26 ± 0.56	1.04 ± 0.46	0.009 *
Climb stairs, M, SD	1.32 ± 0.52	1.14 ± 0.49	0.030 *
Falls, M, SD	0.52 ± 0.65	0.46 ± 0.67	0.620 *

SPPB, short physical performance battery; SARC-F, strength, ambulation, rising from a chair, stair climbing and history of falling; C, COVID-19 patients; NC, non-COVID-19; M patients, mean value; SD, standard deviation value; N, total number; *p* values, statistical significance (*, *t*-test, **, chi-square test).

**Table 5:** Force’s variations according to the patient’s gender.

Parameter	Group C	Group NC	*p*
	Patients’ Number (%)		
Arm Force			
Female, <18 N	0	0	-
Male, <28 N	16 (41.03)	9 (30)	0.161 *
Forearm force			
Female, <18 N	0	1(2.44)	0.890 *
Male, <28 N	26 (66.67)	27 (90)	0.317 *

C, COVID-19 patients; NC, non-COVID-19 patients; N, Newton; n, total number; *p* values, statistical significance (*, chi-square test).

## Data Availability

Data of the patients are available in the medical archive of the Medical Treatment and Rehabilitation Centre, Baile 1 Mai, Ceres Hotel, Bihor County, Romania.
